# Remote Sensing Image Scene Classification with SE-EfficientNetV2-S: An Empirical Study of Channel Attention and Semi-Supervised Pseudo-Labeling

**DOI:** 10.3390/s26144617

**Published:** 2026-07-21

**Authors:** Liting Liao, Haoyuan Yang, Jun Peng, Runqiu Jin

**Affiliations:** 1 School of Computer and Information Engineering, Jiangxi Agricultural University, Nanchang 330045, China; 2 Key Laboratory of Agricultural Information Technology of Higher Education Institutions of Jiangxi Province, Nanchang 330045, China; 3 Nanchang Digital Agriculture and Intelligent Perception Collaborative Innovation Key Laboratory, Nanchang 330045, China

**Keywords:** remote sensing image, scene classification, channel-wise attention mechanism, semi-supervised learning, EfficientNetV2, pseudo-labeling distillation, low-supervision generalization

## Abstract

**Highlights:**

**What are the main findings?**

- A late-stage Squeeze-and-Excitation (SE) channel gating mechanism is integrated after the final 1 × 1 expansion convolution, adaptively recalibrating cross-channel dependencies with negligible parameter overhead (0.2 M); however, multi-seed analysis shows this gain is seed-sensitive and does not reach robust reproducibility at the current dataset scale.
- Under strictly fair fully unfrozen training, the purely supervised SE-EfficientNetV2-S achieves 98.71% test accuracy on a 10-class NWPU-RESISC45 subset, outperforming ResNet50 (98.50%) by 0.21 percentage points at roughly 85% of its parameter count (20.4 M vs. 24.1 M).
- A two-stage semi-supervised pseudo-labeling strategy (τ=0.90) raises validation accuracy to 99.57% and improves mean test accuracy for the No-SE configuration by +0.46 pp across three seeds, while the SE-augmented model shows only a marginal + 0.08 pp gain—indicating that channel gating moderates pseudo-label effectiveness.

**What are the implications of the main findings?**

- Lightweight CNN architectures, when fairly trained, can surpass larger models without requiring semi-supervised augmentation, providing a strong and reproducible supervised baseline for remote sensing scene classification.
- Semi-supervised pseudo-labeling with high-confidence filtering shows a small, sign-consistent improvement for models without channel-wise attention across three seeds (+0.46 pp mean), though not yet statistically significant at this sample size; the effect is smaller still for SE-augmented variants.

**Abstract:**

With the rapid development of remote sensing technology, high-resolution satellite imagery has been increasingly applied to land resource monitoring, urban planning, and environmental assessment. Automatically assigning semantic labels to remote sensing image patches remains a fundamental challenge due to pronounced intra-class variation and high inter-class visual similarity. To address the trade-off between model capacity and limited labeled data, this paper proposes a remote sensing image scene classification framework based on an improved EfficientNetV2-S architecture. The proposed model integrates a Squeeze-and-Excitation (SE) channel attention module between the final 1 × 1 expansion convolution and the Global Average Pooling layer, where it functions as a late-stage channel gating mechanism that adaptively recalibrates channel-wise responses, though its accuracy benefit is seed-sensitive rather than consistently reproducible at the current dataset scale. A two-stage optimization strategy was evaluated, comprising a fully unfrozen supervised baseline followed by a pseudo-label semi-supervised fine-tuning stage utilizing a strict confidence threshold (τ=0.90). Evaluated on a 10-class subset of the public NWPU-RESISC45 benchmark, the purely supervised SE-EfficientNetV2-S delivers 98.71% independent test accuracy, matching or exceeding the much larger ResNet50 (98.50%, 24.1 M parameters) despite using only 20.4 M parameters. Multi-seed variance analysis further reveals that semi-supervised fine-tuning yields a small test-set improvement for the No-SE configuration that is consistent in sign across all three seeds (+0.46 pp mean) but not statistically significant at this sample size, and an even smaller, likewise non-significant gain for the SE-augmented model (+0.08 pp), suggesting that channel gating moderates pseudo-label effectiveness in small-data regimes.

## 1. Introduction

With the rapid growth of high-resolution remote sensing imagery, automatic scene classification has become essential for applications such as urban planning, environmental monitoring, and disaster assessment [1]. Traditional methods relying on hand-crafted features (e.g., SIFT, LBP) often fail to capture the complex semantic content of remote sensing scenes, especially when large intra-class variations and high inter-class similarities exist [2].

Although deep convolutional neural networks (CNNs) have advanced this field [3,4], remote sensing scene classification still faces two interrelated challenges. First, the direct transfer of models pre-trained on standard natural image datasets (e.g., ImageNet) is severely hindered by a pronounced domain gap. This gap stems from the unique characteristics of remote sensing imagery, such as bird’s-eye perspectives, diverse spatial resolutions, and varied seasonal or illumination conditions. Second, acquiring high-quality image-level annotations to bridge this gap is prohibitively expensive and time-consuming, as it heavily relies on specialized expert knowledge. In parallel, attention mechanisms have been widely adopted to enhance feature discrimination by adaptively emphasizing salient regions [5,6]. Lightweight attention-enhanced networks, such as HLAE-Net [7] and dual-layer attention architectures [8], have recently been proposed to balance model complexity and classification accuracy for resource-limited remote sensing applications. Addressing the trade-off between limited labeled datasets and the need for robust feature extraction has made efficiently leveraging abundant unlabeled data—particularly through semi-supervised or self-supervised paradigms [9]—a critical research priority for improving classification performance in low-supervision scenarios.

To address these issues, this paper proposes a method based on an improved EfficientNet and semi-supervised learning. The main contributions are: (1) designing an SE-EfficientNetV2-S model that integrates an SE channel attention module at the pre-pooling location of the lightweight EfficientNetV2-S backbone, functioning as a channel-wise recalibration mechanism to adaptively reweight cross-channel dependencies prior to classification; (2) proposing a two-stage training strategy comprising supervised transfer learning with fully unfrozen parameters followed by a pseudo-label semi-supervised fine-tuning stage to leverage unlabeled data; and (3) demonstrating through systematic experiments—including multi-seed variance analysis, threshold sensitivity testing, and comparisons with multiple baselines—that semi-supervised pseudo-labeling provides consistent test-set gains for the No-SE configuration while the SE-augmented model benefits only marginally, revealing an architecture-dependent interaction between channel gating and pseudo-label effectiveness.

## 2. Materials and Methods

**Figure 1:**
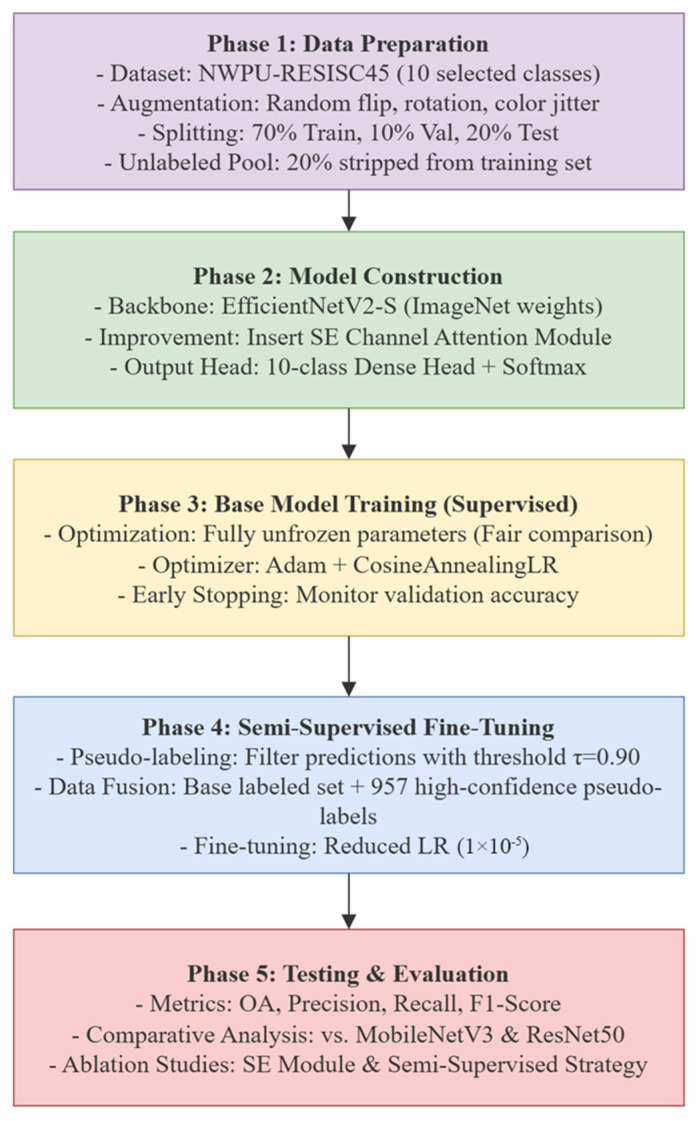
The overall methodology flowchart of the proposed remote sensing image scene classification framework, detailing the five operational phases from data preparation to final evaluation.

To provide a comprehensive overview of the proposed remote sensing image scene classification framework, the overall research methodology is systematically divided into five sequential phases. As illustrated in Figure 1, the pipeline initiates with rigorous data preparation and augmentation (Phase 1), followed by the construction of the SE-EfficientNetV2-S model (Phase 2). The optimization strategy is split into a supervised base model training stage using fully unfrozen parameters (Phase 3) and a subsequent semi-supervised fine-tuning stage that leverages high-confidence pseudo-labels (Phase 4). Finally, the framework undergoes extensive testing, comparative analysis, and ablation studies to validate its generalization capacity (Phase 5).

### 2.1. Dataset Engineering, Selection Criteria, and Preprocessing Tensor Transforms

The experimental data is sourced from the benchmark NWPU-RESISC45 remote sensing dataset released by Northwestern Polytechnical University [10]. In addition to NWPU-RESISC45, several other benchmark datasets such as WH-MAVS [11] and EuroSAT [12] have been widely adopted to evaluate scene classification models under diverse spatial resolutions and geographic coverages. This benchmark repository contains 31,500 independent remote sensing images segmented across 45 unique semantic land-use designations, with each category containing 700 distinct image tiles configured to fixed 256×256 pixel grids. These samples feature wide spatial variation, collected from Google Earth screens across more than 100 countries under varying ambient weather conditions, solar angles, sensor specifications.

**Figure 2:**
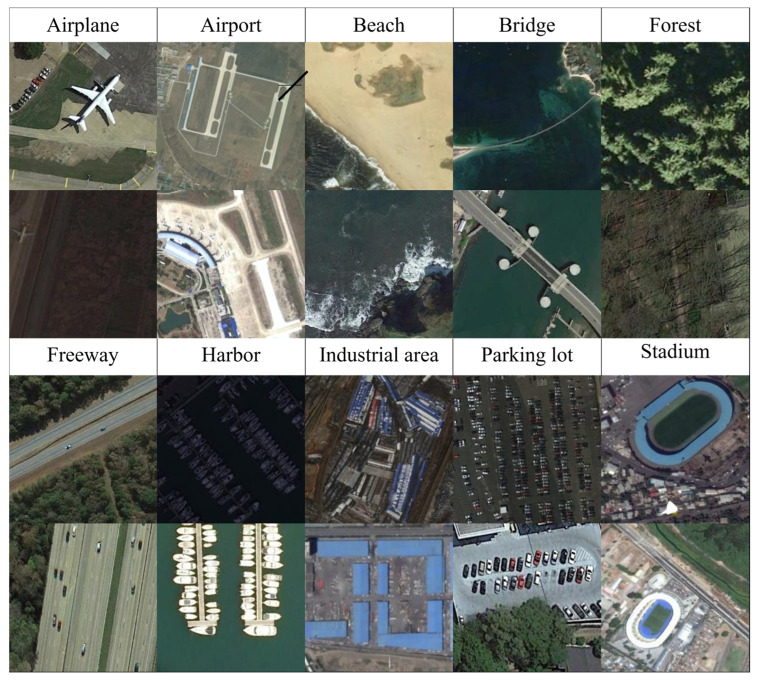
Representative remote sensing images samples across the ten selected semantic land-use categories extracted from the NWPU-RESISC45 benchmark dataset. Rows demonstrate the significant intra-class spatial and seasonal variations within identical categories.

To evaluate the model’s performance on highly confusing land-use categories while maintaining computational efficiency, a 10-class subset containing 7000 distinct image patches was extracted. The ten selected categories include: airplane, airport, beach, bridge, forest, freeway, harbor, industrial area, parking lot, and stadium (as structured and exemplified in Figure 2).

These categories provide an ideal validation environment because they mix distinctive natural topographies (forests, beaches) with structurally complex built environments (airports, industrial areas, harbors) that feature shared geometric elements, such as asphalt strips, concrete slabs, and parallel vehicular patterns. The entire dataset (7000 images) is segmented via an un-skewed 56:10:14:20 ratio. Specifically, we allocated 56% (3920 frames) for supervised initial parameter extraction, 10% (700 frames) for checkpoint early-stopping validations, 14% (980 frames) as the unannotated dataset pool for semi-supervised iterative self-training, and exactly 20% (1400 frames) for the final unbiased evaluation benchmarks. This creates a strictly controlled, in-distribution simulated environment, deliberately designed to isolate and validate the algorithmic efficacy of the pseudo-labeling pipeline prior to future cross-dataset deployments. The structured sample allocations across all classification phases are documented in Table 1.

### 2.2. Data Augmentation and Preprocessing Strategy

**Figure 3:**
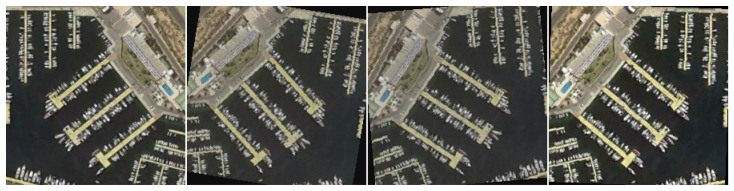
Illustrative examples of the randomized data augmentation pipeline implemented during the supervised baseline initialization stage (demonstrated using the harbor category).

To reduce overfitting under low supervision, labeled training images pass through a randomized augmentation pipeline. Initially, to build resilience against scale variations and partial occlusions, original images undergo a random resized cropping operation. Specifically, sub-regions ranging randomly from 80% to 100% of the original image area are extracted and subsequently resized to a fixed 224 × 224 input tensor. Following this spatial recalibration, images undergo random horizontal mirror reflections (p=0.5), stochastic spatial rotations within a ±10° sweep, and custom color jitter transforms with a ±0.2 deviation factor applied to brightness, contrast, and saturation scales. The visual effects of these tensor transformations are systematically illustrated in Figure 3.

Input frames are resized to a fixed 224×224 matrix layout to align with the model’s structural entry parameters. The data tensor then passes through standard Z-score normalization matching pre-trained ImageNet parameters, as expressed below:

(1) $$T_{\mathrm{normalized}\;}=\frac{T_{\mathrm{raw}}\;-\;\mu_{\mathrm{channel}}}{\sigma_{\mathrm{channel}}},$$

where $\mu_{channel}=\left[0.485,\;0.456,\;0.406\right]$ and $\sigma_{channel}=\left[0.229,\;0.224,\;0.225\right]$. Validation, unlabeled, and test streams strictly skip the augmentation steps, receiving only a deterministic central resize to 224 × 224 followed by the standard ImageNet Z-score normalization.

### 2.3. Mathematical Modeling and Structural Layout of the SE-EfficientNet Model

The structural core of the classification framework utilizes the EfficientNetV2-S topology [13,14]. This network improves upon early compound-scaling convolutional neural networks by replacing standard Mobile Inverted Bottleneck Convolution (MBConv) structures with Fused Mobile Inverted Bottleneck Convolution (Fused-MBConv) modules in its initial stages. The Fused-MBConv module replaces the separate 1×1 expansion convolution and depthwise 3×3 convolution with a single standard 3×3 convolution layer. This structural change reduces memory access latencies and accelerates training velocity without compromising parameter efficiency. The standard 2D convolution operation is expressed as follows:

(2) $$O_{i,j}=\sum_{m=0}^{M-1}\sum_{n=0}^{N-1}I_{i+m,j+n}\cdot K_{m,n}+b,$$

where I represents the incoming feature canvas tensor, K signifies the discrete weight kernel, b is the scalar bias, and $O_{i,j}$ marks the downsampled localization activation point. Non-linear mappings are applied using the Swish activation function ($x\cdot \mathrm{sigmoid}\left(\beta x\right)$).

The core of the EfficientNet family is the compound scaling method, which uniformly scales network depth, width, and input resolution with a set of fixed coefficients. This method is formalized as:

(3) $$\mathrm{depth}:\;d=\alpha^{\phi },\;\mathrm{width}:\;w=\beta^{\phi },\;\mathrm{resolution}:\;r={\;\gamma }^{\phi },$$

where α, β, and γ are constant coefficients determined via a grid search, strictly constrained by the relation $\alpha \cdot \beta^{2}\cdot \gamma^{2}\;\approx \;2$. This foundational principle allows the selected EfficientNetV2-S backbone to achieve an optimal balance between feature representation capacity and parameter efficiency prior to the structural modification of adding the SE block.

To isolate critical target features against complex background land cover, a Squeeze-and-Excitation (SE) block [15] was embedded prior to the final Global Average Pooling (GAP) stage. Specifically, after the Stage 6 MBConv block outputs a 7 × 7 × 256 tensor, a standard 1 × 1 expansion convolution scales the feature dimension to 7 × 7 × 1280. The SE module functions strictly as a late-stage channel gating mechanism. Because the computed scaling factors are spatially invariant across the 7 × 7 grid, this configuration is mathematically equivalent to applying channel-wise recalibration directly to the pooled feature vector, explicitly modeling cross-channel dependencies prior to the classification head without constituting a localized spatial attention mechanism. The SE module explicitly updates cross-channel dependencies via a three-step adaptive recalibration sequence:

Step 1 (Squeeze): The spatial properties of an input feature matrix $U\in R^{H\;\times W\;\times C}$ are compressed across its spatial plane using a Global Average Pooling layer, yielding a channel descriptor vector $z\in R^{1\;\times \;1\;\times \;C}$. The C-th element of this vector is computed as:

(4) $$z_{c}=F_{sq}\left(U_{c}\right)=\frac{1}{H\times W}\sum_{i=1}^{H}\sum_{j=1}^{W}u_{c}\left(i,j\right).$$

Step 2 (Excitation): Cross-channel dependencies are modeled via a non-linear gating bottleneck mechanism configured with a dimensionality reduction ratio *r* = 16. This sequence is defined by:

(5) $$s=F_{ex}\left(z,W\right)=\sigma \left(W_{2}\cdot \delta \left(W_{1}\cdot z\right)\right),$$

where δ represents the ReLU activation operation, σ represents the Sigmoid function, $W_{1}\in R^{\frac{C}{r}\times C}$ denotes the parameter matrix of the downscaling fully connected layer, and $W_{2}\in R^{C\times \frac{C}{r}}$ represents the parameter matrix of the upscaling layer.

Step 3 (Scale): The initial activation maps are scaled through element-wise multiplications against their corresponding channel modulation weights:

(6) $$X_{c}=F_{scale}\left(U_{c},s_{c}\right)=s_{c}\cdot U_{c},$$

where $X_{c}$ denotes the updated feature representation layer passed to the final classification head.

**Figure 4:**
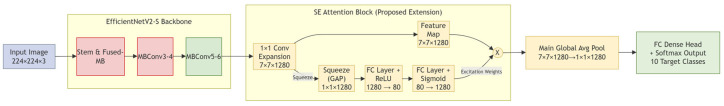
End-to-end operational processing pipeline topology and structural layout of the proposed SE-EfficientNet architecture under fully unfrozen training conditions.

Furthermore, to effectively mitigate the risk of severe overfitting during the optimization process—particularly critical when learning from heavily constrained annotated dataset partitions—a structural Dropout layer is integrated. Positioned immediately after the Global Average Pooling (GAP) stage and prior to the final fully connected dense head, this layer executes stochastic neuron deactivation with a zeroing probability of p=0.3. This ensures the classification head does not develop disproportionate reliance on singular feature channels, forcing the network to learn broadly distributed, highly robust semantic representations. This structural configuration is detailed in Table 2, and the processing pipeline is illustrated in Figure 4.

### 2.4. Dual-Phase Hybrid Semi-Supervised Data Optimization Design

To establish structural stability across the model’s weight matrices under sparse supervision data constraints, a comprehensive two-stage hybrid optimization blueprint is implemented:

Phase 1 (Supervised Parameter Initialization): The network parameters are initialized with pre-trained weights optimized on the ImageNet repository. The added SE module and the dense classification head utilize random initializations. Unlike traditional restrictive freezing strategies, all structural layers are fully unfrozen during this phase. This allows the pre-trained ImageNet weights to act as a robust initialization, while full-parameter gradient backpropagation enables the entire network to rapidly adapt and align with the specific spatial and textural patterns of remote sensing imagery. Training is conducted for 15 epochs with the Adam optimizer and a cosine annealing learning rate schedule, starting from an initial learning rate of 1 × 10^−4^.

Phase 2 (Semi-Supervised Iterative Self-Training): After Phase 1 convergence, the trained base model acts as a pseudo-label generator. Raw images from the unlabeled dataset pool are processed through the network’s inference path to output Softmax class probabilities. Predictions must clear a strict confidence threshold (τ=0.90) to qualify as accurate designations:

(7) $$y_{n}=\arg\underset{y}{\max}P\left(y|x_{n},\theta_{base}\right),\;\mathrm{subject}\;\mathrm{to}\;\max P\left(y|x_{n},\theta_{base}\right)\geq 0.9.$$

Images that meet this criterion are assigned a deterministic categorical pseudo-label. Due to the strict nature of the τ=0.90 threshold, the yield across different categories was slightly asymmetrical (totaling 957 samples rather than the theoretical maximum of 980). We intentionally prioritized the absolute confidence of the pseudo-labels over forced class balancing to prevent the introduction of confirmation noise. Full-parameter gradient backpropagation is continued at a reduced fine-tuning learning rate (1 × 10^−5^) using an early-stopping monitor. The proposed framework was implemented using the PyTorch deep learning library [16]. The software version is 1.12.1 with CUDA 11.3 computing platform. The training hyperparameters are specified in Table 3.

### 2.5. Evaluation Metrics

To quantitatively evaluate the classification performance of the proposed model, four standard metrics are employed: Overall Accuracy (OA), Precision, Recall, and the F1-Score. Let TP, TN, FP, and FN represent the true positives, true negatives, false positives, and false negatives, respectively. The evaluation metrics are defined as follows:

(8) $$Accuracy=\frac{TP+TN}{TP+TN+FP+FN},$$

(9) $$Precision=\frac{TP}{TP+FP},$$

(10) $$Recall=\frac{TP}{TP+FN},$$

(11) $$F1=\frac{2\times Precision\times Recall}{Precision+Recall}.$$

For the 10-class multi-classification task, macro-averaged and weighted-averaged formulations of the F1-Score are applied to ensure an unbiased performance assessment across potentially challenging subsets.

### 2.6. Transfer Learning

Training a deep convolutional neural network from scratch typically requires a massive amount of labeled data. Transfer learning addresses this by taking a model pre-trained on a large-scale source dataset (e.g., ImageNet) and fine-tuning it on the target dataset [17], a strategy particularly effective for remote sensing, where labeled data is scarce, since low-level features (e.g., edges, textures) are often generalizable across domains.

The SE-EfficientNet is initialized with ImageNet pre-trained weights. Rather than using a restrictive layer-wise freezing strategy or a linear probe that trains only the classifier, the entire network is fully unfrozen during training, allowing it to overcome the domain gap between natural, object-centric images and overhead geographic scenes while mitigating underfitting. This strategy has been successfully applied in other remote sensing tasks, including hyperspectral image classification with residual dense connections [18] and few-shot fine-tuning frameworks such as TA-MSA [19].

Because labeled data is limited, the framework also leverages unannotated samples through high-confidence pseudo-labeling (τ = 0.90) to expand the effective training distribution, reducing reliance on large labeled datasets. As demonstrated in Section 3.6, this strategy shows a small, sign-consistent test-set gain for the No-SE configuration across three seeds that has not reached statistical significance at this scale, while its benefit for the SE-augmented model is more modest still.

### 2.7. Pseudo-Label Semi-Supervised Learning

Semi-supervised learning aims to improve model performance by leveraging unlabeled data alongside a small set of labeled data. Pseudo-labeling is one of the simplest and most effective methods [20]. The process involves three steps. First, a “base model” is trained exclusively on the labeled data. Second, this base model is used to predict labels for the unlabeled data. Predictions with a maximum class probability (confidence) exceeding a predefined threshold τ are accepted as “pseudo-labels”. Third, these high-confidence pseudo-labeled samples are combined with the original labeled dataset to form an augmented training set, on which the model is re-trained or fine-tuned. This iterative process allows the model to learn from its own most confident predictions, effectively exploiting the underlying data distribution of the unlabeled samples. The confidence threshold τ is a critical hyperparameter; a high threshold ensures high-quality pseudo-labels but yields few samples, while a low threshold may introduce noise.

## 3. Results

### 3.1. Detailed Empirical Analysis of Optimization Convergence Dynamics

Under the fully unfrozen supervised configuration (Phase 1), the SE-EfficientNetV2-S model demonstrated rapid convergence, reaching 98.57% validation accuracy after only one epoch—confirming the strong transfer capability of ImageNet pre-trained weights. As detailed in Table 4, the validation accuracy stabilized around 99.00–99.14% from Epoch 3 onward, peaking at 99.14% at Epochs 9, 11, and 13, with training accuracy continuing to rise to 99.85% by Epoch 15 without signs of validation degradation. This divergence between training and validation accuracy in later epochs is expected under cosine annealing scheduling and does not indicate overfitting, as the best checkpoint is retained via early stopping on the validation set.

**Figure 5:**
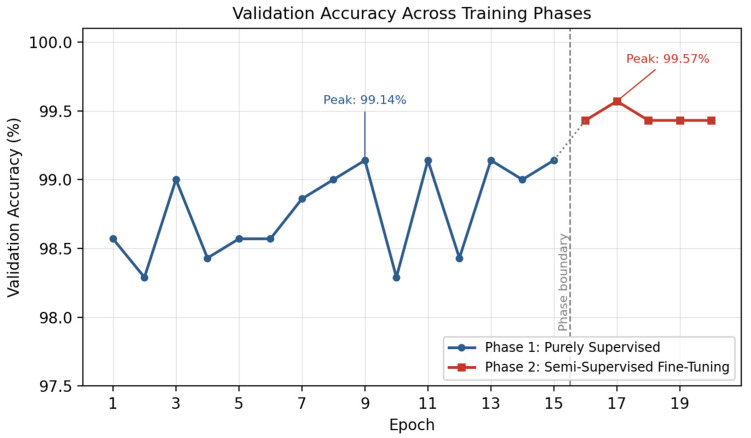
Validation accuracy progression across Phase 1 (purely supervised, Epochs 1–15) and Phase 2 (semi-supervised pseudo-label fine-tuning, Epochs 16–20).

During Phase 2 (Semi-Supervised Fine-Tuning), a sensitivity analysis was conducted to determine the optimal pseudo-labeling confidence threshold (τ). Empirical testing at τ = 0.80 and τ = 0.95 yielded peak validation accuracies of 99.43%. Applying the optimal τ = 0.90 confidence filter extracted 957 high-confidence pseudo-labels from the unannotated pool, with a slightly asymmetrical class distribution across categories. To prevent the introduction of confirmation bias, absolute confidence was prioritized over forced strict class balancing. A maximum extraction cap of 97 images per category was instituted. The strict τ=0.90 threshold yielded a slightly asymmetrical but highly balanced distribution totaling 957 samples, indicating that the baseline model exhibits strong and relatively uniform prediction confidence across all spatial topologies. The precise class-wise distribution of the extracted pseudo-labels is documented in Table 5. Fine-tuning on this augmented dataset elevated the internal validation accuracy to a peak of 99.57%. The validation accuracy progression across both training phases is illustrated in Figure 5, showing the transition from a more variable Phase 1 trajectory (98.29–99.14%) to a consistently higher and more stable Phase 2 plateau (99.43–99.57%) following the introduction of pseudo-labeled samples.

### 3.2. Quantitative Generalization Assessment on the Independent Test Set

The optimized models were evaluated on the independent 1400-image test set. Under semi-supervised fine-tuning, the SE-EfficientNetV2-S achieved 98.86% overall test accuracy (seed 2026), compared to 98.71% under purely supervised training, confirming that pseudo-label augmentation provides a modest but positive generalization benefit for this configuration. The global evaluation metrics for the semi-supervised SE-EfficientNetV2-S are detailed in Table 6. Because the test set is perfectly balanced (140 images/category), macro- and weighted-average metrics approximate overall accuracy; the small deviation of macro precision (98.88%) from overall accuracy (98.86%) reflects minor asymmetries in per-class precision distributions.

### 3.3. Per-Class Diagnostic Performance and Error Root Analysis

**Figure 6:**
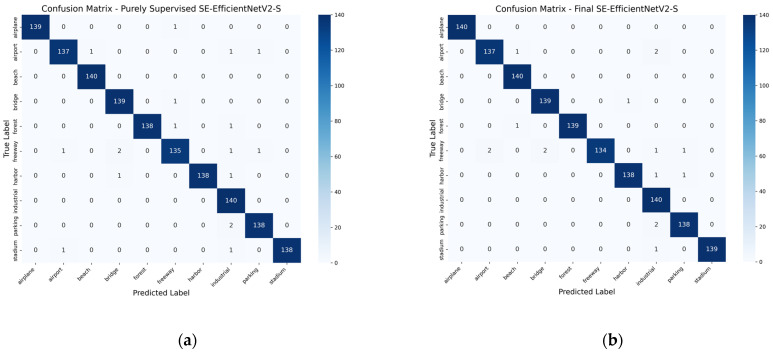
(**a**) The confusion matrices on the independent 1400-image test set for the purely supervised SE-EfficientNetV2-S; (**b**) the confusion matrices on the independent 1400-image test set for the semi-supervised fine-tuned variant. Off-diagonal entries indicate per-class misclassification counts.

Class-specific precision, recall, and F1-Scores for the semi-supervised SE-EfficientNetV2-S (seed 2026) are summarized in Table 7. Section 3.6 extends this single-run diagnostic with a three-seed variance analysis to assess the stability of these per-class scores. The model maintains strong classification performance across all ten categories. Categories with distinctive structural topologies—specifically airplane, forest, freeway, and stadium—achieved perfect precision scores of 100.0%. The lowest-performing category is industrial area, with an F1-Score of 97.56%, primarily due to geometric overlap with harbor and parking lot patterns. Figure 6 compares the test-set confusion matrices for the purely supervised and semi-supervised configurations side-by-side, showing that failure modes remain largely consistent across both training variants.

### 3.4. Comparative Evaluation Across Alternative Architectures

To comprehensively address potential architectural confounds and ensure a strictly fair empirical comparison, all evaluated models—including the proposed SE-EfficientNetV2-S, MobileNetV3-Small [21], and ResNet50 [4]—were trained under fully unfrozen conditions. By avoiding aggressive layer-wise freezing, all networks could freely adapt their pre-trained ImageNet representations to the remote sensing domain.

To ensure a fair comparison, ResNet50 was augmented with an SE attention block inserted after the final residual stage (layer4) and prior to the Global Average Pooling layer, following the same pre-pooling placement principle as the proposed SE-EfficientNetV2-S. The SE module uses a reduction ratio of r = 16, consistent with the proposed model. This configuration is referred to as ResNet50 + SE throughout the paper.

As shown in Table 8, the proposed SE-EfficientNetV2-S achieved 98.71% independent test accuracy under pure supervision, outperforming ResNet50 (98.50%) while requiring approximately 15% fewer parameters (20.4 M vs. 24.1 M). MobileNetV3-Small achieved 97.14% test accuracy despite its extremely compact 1.5 M parameter footprint. Under semi-supervised fine-tuning, the No-SE EfficientNetV2-S configuration achieves the highest test accuracy across all evaluated models (99.36%, seed 2026), while the SE-augmented variant reaches 98.86%—both surpassing their purely supervised counterparts on this seed; see Section 3.6 for the multi-seed significance analysis of this effect.

### 3.5. Component-Level Ablation Study

To isolate the individual performance contributions of the SE channel-wise attention block and the semi-supervised self-training logic, a four-way ablation was conducted under identical dataset partitions. Results are detailed in Table 9 (single-run, seed 2026).

The baseline configuration (Group 4: No-SE, No SSL) achieved 98.50% test accuracy. Integrating the SE attention block under pure supervision (Group 3) increased test accuracy to 98.71%, a +0.21 pp gain at negligible parameter overhead. Applying semi-supervised fine-tuning to the No-SE backbone (Group 2) raised test accuracy further to 99.36%, demonstrating that pseudo-label augmentation provides a substantial generalization benefit when channel gating is absent. The full synthesis (Group 1: SE + SSL) achieved 98.86% test accuracy—higher than the purely supervised SE baseline (98.71%) but lower than the semi-supervised No-SE configuration (99.36%), suggesting that the SE gating mechanism partially moderates the effectiveness of pseudo-label augmentation. Internal validation accuracies peaked at 99.43% and 99.57% for Groups 2 and 1 respectively, confirming that the pseudo-labeled samples carry genuine signal. The gap between validation and test gains is more pronounced for the SE configuration, consistent with the multi-seed findings in Section 3.6.

### 3.6. Multi-Seed Variance Analysis

The ablation results in Table 9 reveal several important findings. First, under purely supervised training, the SE module provides a positive single-seed gain: the SE-augmented model (Group 3) achieves 98.71% test accuracy, surpassing the No-SE baseline (Group 4) at 98.50%—a +0.21 pp improvement with negligible parameter overhead (~0.2 M additional parameters).

Second, semi-supervised pseudo-labeling improves test accuracy for both configurations. For the No-SE backbone (Group 4 → Group 2), test accuracy rises from 98.50% to 99.36% alongside a validation gain from 99.29% to 99.43%, indicating that pseudo-labels carry genuine generalization value when channel gating is absent. For the SE-augmented model (Group 3 → Group 1), the test accuracy improvement is more modest (98.71% → 98.86%), despite a larger validation gain (99.14% → 99.57%). This asymmetry suggests that the SE gating mechanism partially moderates the effectiveness of pseudo-label augmentation, absorbing less of the additional training signal than the No-SE backbone.

To verify the reproducibility of the purely supervised SE gain (+0.21 pp, Table 9) beyond a single seed, Group 3 and Group 4 were re-evaluated across three independent random seeds (1024, 2026, and 3038). As shown in Table 10, the SE-augmented model outperforms the No-SE baseline on two seeds (1024: 98.93% vs. 98.79%; 2026: 98.71% vs. 98.50%) but underperforms on the third (3038: 98.50% vs. 98.86%). Averaged across seeds, both configurations converge to identical mean test accuracy (No-SE: 98.71% ± 0.15%; SE: 98.71% ± 0.17%), indicating that the single-seed advantage falls within normal seed-to-seed variance.

Table 11 details per-class F1-Scores across three seeds under the semi-supervised configuration. The No-SE baseline achieves higher mean F1-Scores and lower variance than the SE model across most categories, most notably in forest (No-SE: ±0.45 pp; SE: ±0.79 pp) and industrial area (No-SE: ±0.33 pp; SE: ±0.71 pp). This category-level volatility is consistent with the hypothesis that the SE gating mechanism amplifies the effect of noisy pseudo-labels in complex semantic categories.

In summary, the multi-seed evidence shows a positive test-set effect for the No-SE configuration that is consistent in sign across all three seeds (+0.21, +0.86, +0.28 pp; mean + 0.45 pp ± 0.36 pp), though a paired *t*-test (t(2) ≈ 2.2, *p* ≈ 0.16) indicates this has not yet reached statistical significance. The benefit for the SE-augmented model is smaller still (+0.08 pp) and likewise not statistically established. Larger-sample replication would be needed to confirm either effect at conventional significance levels.

### 3.7. Impact of Labeled Data Volume

To further investigate the data efficiency of the proposed framework, a supplementary labeled-volume ablation was conducted. The fully unfrozen supervised SE-EfficientNetV2-S model was re-trained using 25%, 50%, and 100% of the available supervised training partition (corresponding to 980, 1960, and 3920 images, respectively).

As detailed in Table 12, the independent test accuracy remained within a tight margin, achieving 98.50% with only a quarter of the training data, and peaking at 98.71% with the full labeled set. This non-monotonic ~0.5 percentage point variance falls within the natural seed-to-seed fluctuation bounds established in Section 3.6. These preliminary results suggest that the robust ImageNet-pretrained backbone allows the model to approach its performance ceiling on this 10-class subset with as little as 25% of the labeled data, providing a highly data-efficient baseline for remote sensing applications.

## 4. Discussion

### 4.1. Effects of Semi-Supervised Pseudo-Labeling on Test-Set Generalization

Multi-seed analysis (Table 10) reveals that semi-supervised pseudo-labeling provides a genuine improvement in independent test accuracy for the No-SE configuration: mean test accuracy rises from 98.71% ± 0.15% under pure supervision to 99.17% ± 0.15% under semi-supervised fine-tuning—a +0.46 pp mean gain that is consistent in sign across all three seeds (1024: +0.21 pp; 2026: +0.86 pp; 3038: +0.28 pp; mean + 0.45 pp ± 0.36 pp). A paired *t*-test across the three seeds (t(2) ≈ 2.2, *p* ≈ 0.16) indicates that, as with the SE and ResNet50 comparisons discussed in Section 4.2 and Section 4.4, this effect has not been established as statistically significant at the current sample size. This result is consistent with the single-seed observation in Table 9 (seed 2026: No-SE semi-supervised = 99.36%); taken together, the evidence points to a small, sign-consistent improvement for the No-SE backbone rather than a firmly established generalization gain.

For the SE-augmented model, the benefit is substantially more modest: mean semi-supervised test accuracy is 98.79% ± 0.06%, compared to 98.71% ± 0.17% under pure supervision—a marginal +0.08 pp difference that falls within the range of seed-to-seed variance. The No-SE semi-supervised configuration therefore achieves a higher mean test accuracy (99.17%) than the SE semi-supervised configuration (98.79%), reversing the ordering observed under purely supervised training, where both converged to 98.71%.

We attribute this asymmetry to the interaction between the SE gating mechanism and pseudo-label quality. Without channel-wise attention, the model updates its weights more uniformly in response to the pseudo-labeled data, absorbing the additional training signal without selectively suppressing certain feature channels. The SE module, by contrast, adaptively scales channel responses based on learned dependencies; when pseudo-labels introduce even mild confirmation bias, this gating mechanism can amplify spurious correlations in the noisy pseudo-labeled samples—leading to the higher per-class variance observed in Table 11 for complex categories such as forest (SE: ±0.79 pp vs. No-SE: ±0.45 pp) and industrial area (SE: ±0.71 pp vs. No-SE: ±0.33 pp).

The internal validation accuracy improved substantially for both configurations under semi-supervised fine-tuning (No-SE: → 99.43%; SE: → 99.57%), confirming that the pseudo-labeled samples carry informative signals. The smaller test-set gain relative to the validation gain reflects the in-distribution nature of the unlabeled pool—pseudo-labels reinforce the model’s existing decision boundaries but provide limited exposure to novel variations—rather than indicating overfitting in the classical sense. These findings suggest that validation accuracy alone remains a useful but incomplete indicator of generalization when the unlabeled pool is drawn from the same distribution as the training data.

### 4.2. The SE Module Under Pure Supervision: A Seed-Sensitive, Not Consistently Reproducible Gain

Under purely supervised training on the seed reported in Table 9, the SE module contributed a +0.21 pp improvement in test accuracy (98.50% → 98.71%) with only 0.2 M additional parameters, a magnitude consistent with gains of 0.5–1.5% typically reported in the original SE literature. However, multi-seed analysis (Table 10) shows this advantage does not hold consistently: across three independent seeds, the SE-augmented model outperforms the no-SE baseline on two seeds but underperforms on the third, and the two configurations converge to an identical mean test accuracy (98.71% ± 0.15–0.17%). This indicates that the SE module’s benefit under pure supervision, while real on certain initializations, is of a magnitude comparable to inherent seed-to-seed variance and should not be characterized as a consistently reproducible gain at the current dataset scale. Under semi-supervised settings, the SE-augmented model showed slightly lower mean test accuracy than its no-SE counterpart, and exhibited notably higher per-class F1-Scores variance in complex semantic categories such as forest and industrial area. Specifically, because the SE module adaptively scales feature channels based on learned dependencies, it is highly sensitive to the confirmation bias introduced by low-quality pseudo-labels. In small-data regimes, this gating mechanism may inadvertently amplify spurious correlations present in the noisy pseudo-labels, leading to the more modest and less consistent test-set gains observed for the SE-augmented model under semi-supervised fine-tuning.

### 4.3. Comparison with Alternative Architectures

As summarized in Table 8, the proposed SE-EfficientNetV2-S reached 98.71% test accuracy under pure supervision with 20.4 M parameters—a 0.21 pp margin over the 24.1 M-parameter ResNet50 (98.50%) at roughly 15% fewer parameters. Under semi-supervised fine-tuning, the No-SE EfficientNetV2-S configuration achieves 99.36% (seed 2026), representing the strongest single-run result across all evaluated configurations. MobileNetV3-Small (1.5 M, 97.14%) trails considerably in accuracy but remains an attractive option for severely resource-constrained deployments. The absence of ViT/Swin comparisons is a limitation driven by GPU memory constraints (4 GB VRAM).

Recent studies have introduced sophisticated architectures to the remote sensing domain, such as transformer-based heterogeneously salient graph representations [22], image-to-pixel representation for weakly supervised hyperspectral classification [23], and frameworks for overcoming feature incompleteness [24]. While these methods achieve strong performance, they typically incur significant computational and memory costs. The proposed framework prioritizes lightweight efficiency: the SE channel attention module functions as a channel-wise recalibration mechanism prior to Global Average Pooling, introducing only 0.2 M additional parameters while adaptively reweighting cross-channel dependencies. This makes the framework practical for deployment on resource-constrained platforms such as 4 GB VRAM GPUs or CPU-only environments.

### 4.4. Limitations

Several limitations of this study should be acknowledged. First, to ensure strictly fair, fully unfrozen comparisons, all experiments were conducted under strict hardware memory constraints (a 4 GB VRAM GPU). This required small batch sizes (Batch Size = 4) with gradient accumulation to simulate a larger effective batch size. While this ensured mathematical equivalence, future work will leverage high-performance computing clusters to scale this framework across the complete 45-class NWPU-RESISC45 dataset, as well as other global benchmarks like AID or UC Merced.

Second, the confidence threshold τ=0.90 was selected based on single-seed sensitivity analysis comparing τ=0.80 and τ=0.95, where all three thresholds produced validation accuracy differences of only 0.14 pp—equivalent to a single image on the 700-image validation set. This margin is too narrow to support strong claims about threshold optimality. In practice, τ=0.90 extracted 957 of 980 unlabeled samples (97.7%), with a slightly asymmetrical class distribution. For deployment on noisier or cross-domain remote sensing datasets, a dynamic thresholding strategy (e.g., FlexMatch [25]) would likely be required to handle varying confidence levels across semantic categories.

Third, due to the aforementioned hardware constraints, comparisons were not made with recent vision transformers (e.g., Swin, ViT), which have shown promising results in remote sensing scene classification. Comparing the SE-EfficientNetV2-S with recent lightweight transformer-based models, such as the dual-branch Swin Transformer [26], would provide a more comprehensive efficiency-accuracy benchmark in future work.

Fourth, the + 0.21 pp improvement attributed to the SE module under pure supervision (Table 9) was obtained from a single random seed; multi-seed evaluation (Table 10) shows this gain falls within normal seed-to-seed variance, with mean test accuracy tying at 98.71% for both configurations across three seeds. This means the SE module’s benefit under pure supervision, while architecturally cheap, has not been established as statistically robust at the current 10-class, single-split dataset scale. The same caveat applies to the cross-architecture comparison against ResNet50 (Table 8): the reported 0.21 pp margin is also based on a single seed and has not undergone equivalent multi-seed verification. Larger-scale, multi-seed evaluation—covering both the internal SE ablation and the cross-architecture baselines—would be required to confirm whether either effect generalizes.

## 5. Conclusions

This paper presented an SE-EfficientNetV2-S framework with two-stage semi-supervised training evaluated on a 10-class NWPU-RESISC45 subset. Key conclusions are as follows.

The purely supervised SE-EfficientNetV2-S establishes a strong and competitive lightweight baseline. With 20.4 M parameters and 98.71% independent test accuracy, it surpasses the considerably larger ResNet50 (98.50%, 24.1 M parameters) at roughly 85% of its parameter count. This result demonstrates that careful training protocol—specifically, fully unfrozen fine-tuning from ImageNet pre-trained weights without restrictive layer-wise freezing—rather than sheer model size or architectural complexity, is the primary driver of strong performance on fine-grained remote sensing scene classification. The proposed framework thus provides a robust and reproducible supervised baseline for resource-constrained deployment scenarios.

SE channel attention shows a seed-sensitive gain that does not reach robust reproducibility at the current dataset scale. On the reported seed, the SE module contributed a +0.21 pp improvement in test accuracy (98.50% → 98.71%) with negligible parameter overhead (~0.2 M additional parameters). However, multi-seed evaluation across three independent initializations shows this advantage reverses on one seed and disappears entirely in the cross-seed mean, with both SE and No-SE configurations converging to 98.71% ± 0.15–0.17%. The SE module’s accuracy benefit under pure supervision should therefore be treated as an initialization-sensitive effect rather than a consistently reproducible architectural advantage at the current 10-class, single-split dataset scale.

Semi-supervised pseudo-labeling shows a small, architecture-dependent test-set effect. Across three independent seeds, the No-SE configuration gains a mean + 0.45 pp (±0.36 pp) in test accuracy under semi-supervised fine-tuning (98.71% → 99.17%), consistent in sign across all three seeds but not statistically significant at this sample size (paired t(2) ≈ 2.2, *p* ≈ 0.16). For the SE-augmented model, the corresponding gain is marginal (+0.08 pp; 98.71% → 98.79%), indicating that the channel gating mechanism moderates the effectiveness of pseudo-label augmentation. Validation accuracy improved substantially for both configurations (→99.43% and 99.57%, respectively), but the larger test gains for the No-SE variant suggest that validation metrics alone should not be treated as the sole indicator of generalization when the unlabeled pool is drawn from the same distribution as the training data.

Multi-seed analysis reveals that SE and semi-supervision interact in category-specific ways. Under semi-supervised fine-tuning, the No-SE configuration achieves both a higher mean overall test accuracy (99.17% vs. 98.79%) and lower per-class variance than the SE-augmented model across most categories—most notably in forest (No-SE: ±0.45 pp; SE: ±0.79 pp) and industrial area (No-SE: ±0.33 pp; SE: ±0.71 pp). This suggests that the SE gating mechanism, while beneficial under clean supervised training, amplifies the effect of incorrect pseudo-labels in small-data regimes. Understanding whether this instability stems from overconfident channel suppression or from the gating network memorizing spurious correlations in pseudo-labeled samples remains an open question for future research.

Future work directions: Subsequent research will scale this framework to complete benchmark datasets and evaluate generalization across independent cross-domain sources. Adaptive thresholding strategies (e.g., FlexMatch [25], FixMatch [27]) will be integrated to dynamically manage varying confidence levels across different semantic categories. Furthermore, head-to-head comparisons with modern lightweight vision transformers [26] and the application of model compression techniques will be pursued to fully optimize real-time deployment on severely resource-constrained on-board edge processors.

## Figures and Tables

**Table 1:** Dataset split distribution and operational purpose. All splits are drawn from the 10-class NWPU-RESISC45 subset following a 56:10:14:20 ratio.

Dataset Split	Images Per Class	Total Images	Purpose
Training Set	392	3920	Supervised baseline training
Validation Set	70	700	Hyperparameter tuning and early stopping
Unlabeled Pool	98	980	Pseudo-label generation
Test Set	140	1400	Independent generalization evaluation

**Table 2:** Structural stage parameters of the proposed SE-EfficientNet classification framework.

Layer/Stage Designation	Output Matrix Dimension	Operational Module Specification & Block Configurations
Input Tensor	224 × 224 × 3	Standard RGB remote sensing patch sample vector
Stem Conv Layer	112 × 112 × 24	Initial 3 × 3 standard convolutional processing step, Stride = 2
Stage 1 (Fused-MBConv)	56 × 56 × 24	Fused Mobile Bottleneck module, parameter expansion factor = 1
Stage 2 (Fused-MBConv)	28 × 28 × 48	Fused Mobile Bottleneck module, parameter expansion factor = 4, Stride = 2
Stage 3 (MBConv Block)	14 × 14 × 64	Standard Inverted Bottleneck module, expansion factor = 4, Stride = 2
Stage 4 (MBConv Block)	14 × 14 × 128	Standard Inverted Bottleneck module, expansion factor = 4, Stride = 1
Stage 5 (MBConv Block)	7 × 7 × 160	Standard Inverted Bottleneck module, expansion factor = 6, Stride = 2
Stage 6 (MBConv Block)	7 × 7 × 256	Standard Inverted Bottleneck module, expansion factor = 6, Stride = 1
1 × 1 Conv (Channel Expansion)	7 × 7 × 1280	Dimensionality expansion before pooling
SE Attention Block	7 × 7 × 1280	Adaptive channel-wise dependency recalibration (r = 16)
Global Average Pool	1 × 1 × 1280	Spatial dimension compression via global flattening operations
Dropout Layer	1 × 1 × 1280	Stochastic neuron deactivation (*p* = 0.3) for regularization
Linear Dense Head	10	Fully connected linear mapping layer producing Cross-Entropy logits

**Table 3:** Detailed discrete hyperparameter configurations for distinct operational optimization phases.

Optimization Hyperparameter Designation	Phase 1 Base Labeled Supervised Training	Phase 2 Full Parameter Semi-Supervised Fine-Tuning
Batch Size	8 Samples per Batch Cycle	8 Samples per Batch Cycle
Max Epochs	15 Full Dataset Iterations	5 Full Dataset Iterations
Initial Learning Rate	1 × 10^−4^	1 × 10^−5^
Optimizer	Adam Optimizer Core	Adam Optimizer Core
Weight Decay Scale Regularization	1 × 10^−4^	1 × 10^−4^
Weight Decay	CosineAnnealingLR Pathway	CosineAnnealingLR Pathway
Loss Function	Standard Categorical Cross-Entropy	Standard Categorical Cross-Entropy

**Table 4:** Metric progression tracking across supervised Phase 1 baseline parameter optimization loops.

Training Loop Index	Training Loss	Training Accuracy (%)	Validation Checkpoint Accuracy (%)
Epoch 1	0.8460	78.52	98.57
Epoch 2	0.2488	93.24	98.29
Epoch 3	0.1732	95.18	99.00
Epoch 4	0.1143	97.12	98.43
Epoch 5	0.0945	97.42	98.57
Epoch 6	0.0878	97.73	98.57
Epoch 7	0.0609	98.34	98.86
Epoch 8	0.0421	98.88	99.00
Epoch 9 (Peak)	0.0350	99.11	99.14
Epoch 10	0.0267	99.41	98.29
Epoch 11 (Peak)	0.0197	99.54	99.14
Epoch 12	0.0154	99.64	98.43
Epoch 13 (Peak)	0.0157	99.67	99.14
Epoch 14	0.0152	99.72	99.00
Epoch 15	0.0079	99.85	99.14

**Table 5:** Per-class distribution of the 957 high-confidence pseudo-labels extracted during the semi-supervised stage (Threshold τ=0.90, Max Cap = 97).

Category	Extracted Pseudo-Labels
Airplane	96
Airport	91
Beach	97
Bridge	96
Forest	97
Freeway	94
Harbor	97
Industrial Area	96
Parking Lot	96
Stadium	97

**Table 6:** Consolidated global evaluation metrics on the independent test set for the semi-supervised SE-EfficientNetV2-S (seed 2026).

Metric	Value (%)
Overall Accuracy (OA)	98.86
Macro-Average Precision Score	98.88
Macro-Average Recall Score	98.86
Macro-Average Balanced F1-Score	98.86
Weighted-Average Balanced F1-Score	98.86

**Table 7:** Granular category classification breakdowns across the test dataset.

Target Geographic Class	Precision (%)	Recall (%)	Computed F1-Score (%)	Support
Airplane	100.00	100.00	100.00	140
Airport	98.56	97.86	98.21	140
Beach	98.59	100.00	99.29	140
Bridge	98.58	99.29	98.93	140
Forest	100.00	99.29	99.64	140
Freeway	100.00	95.71	97.81	140
Harbor	99.28	98.57	98.92	140
Industrial Area	95.24	100.00	97.56	140
Parking Lot	98.57	98.57	98.57	140
Stadium	100.00	99.29	99.64	140

**Table 8:** Test accuracy comparison across convolutional backbone architectures under strictly fair, fully unfrozen training conditions. Semi-supervised test accuracy results correspond to seed 2026. The best results are shown in bold.

Model	Parameters	Supervised Test Accuracy	Semi-Supervised Test Acc
MobileNetV3-Small	1.5 M	97.14%	\
ResNet50 + SE Block	24.1 M	98.50%	\
EffNetV2-S (No-SE)	20.2 M	98.50%	**99.36%**
EfficientNetV2-S + SE (Proposed)	20.4 M	**98.71%**	98.86%

**Table 9:** Component-level ablation results on the independent test set. All configurations use the same dataset split and fully unfrozen training protocol. Single-run results correspond to seed 2026. The best results are shown in bold.

Experimental Configuration	SE Attention Block	Semi-Supervised Module	Peak Validation Accuracy (%)	Final Test Dataset Accuracy (%)
Group 1 (Proposed Synthesis)	Yes	Yes	**99.57**	98.86
Group 2 (No Attention Core)	No	Yes	99.43	**9** **9.36**
Group 3 (SE Only (No SSL))	Yes	No	99.14	98.71
Group 4 (Baseline (No-SE, No SSL))	No	No	99.29	98.50

**Table 10:** Overall test accuracy (Mean ± Standard Deviation) for the purely supervised configurations (Group 3 vs. Group 4) evaluated across three independent random seeds (1024, 2026, and 3038). The best results are shown in bold.

Seed	No-SE SSL	SE SSL	No-SE Supervised	SE Supervised
1024	99.00%	98.71%	98.79%	**98.93%**
2026	**99.36%**	**98.86%**	98.50%	98.71%
3038	99.14%	98.79%	**98.86%**	98.50%
Mean ± Std	99.17% ± 0.15%	98.79% ± 0.06%	98.71% ± 0.15%	98.71% ± 0.17%

**Table 11:** Per-class F1-Scores (Mean ± Standard Deviation) evaluated across three independent random seeds (1024, 2026, and 3038) under the semi-supervised fine-tuning configuration. The best results are shown in bold.

Category	No-SE Baseline (%)	SE-EfficientNet (%)
Airplane	**99.763 ± 0.205**	99.644 ± 0.355
Airport	**98.338 ± 0.203**	98.215 ± 0.332
Beach	**99.409 ± 0.204**	99.407 ± 0.205
Bridge	**99.165 ± 0.208**	98.684 ± 0.424
Forest	**99.399 ± 0.551**	98.913 ± 0.971
Freeway	**98.320 ± 0.217**	97.371 ± 0.383
Harbor	**99.165 ± 0.208**	98.810 ± 0.206
Industrial Area	**99.175 ± 0.407**	98.358 ± 0.867
Parking Lot	**99.286 ± 0.356**	98.812 ± 0.208
Stadium	**99.642 ± 0.000**	99.644 ± 0.355

**Table 12:** Supervised test accuracy across varying labeled training data volumes (evaluated on a single random seed).

Labeled Training Volume	Number of Images	Final Test Accuracy (%)
25%	980	98.50
50%	1960	98.21
100%	3920	98.71

## Data Availability

The original NWPU-RESISC45 dataset used in this study is publicly available at https://gcheng-nwpu.github.io/#Datasets (accessed on 1 March 2026). To ensure strict reproducibility, the pre-trained model weights, and the exact 10-class dataset splits utilized in our experiments have been deposited in a public repository and are openly available on Zenodo at https://doi.org/10.5281/zenodo.20995485. The code is publicly available in the repository at https://github.com/lottie7charlotte/SE-EfficientNet-Semi-Supervised (accessed on 1 July 2026).

## Abbreviations

The following abbreviations are used in this manuscript:

CNN	Convolutional Neural Network
SE	Squeeze-and-Excitation
